# Molecular characterization of a galactomannan extracted from Tara (*Caesalpinia spinosa*) seeds

**DOI:** 10.1038/s41598-023-49149-3

**Published:** 2023-12-11

**Authors:** Gabriela Ibieta, Atma-Sol Bustos, Jimena Ortiz-Sempértegui, Javier A. Linares-Pastén, J. Mauricio Peñarrieta

**Affiliations:** 1 Biotechnology, Faculty of Engineering LTH, Lund University, PO Box 117, 221 00 Lund, Sweden; 2 Instituto de Investigaciones Químicas IIQ, Universidad Mayor de San Andrés UMSA, Av. Villazón N° 1995, 0201-0220 La Paz, Bolivia

**Keywords:** Chemistry, Engineering

## Abstract

Tara gum (TG) is a polysaccharide extracted from the seeds of a South American tree called Tara (*Caesalpinia spinosa*). TG is a galactomannan with many applications in the food industry, mainly as an emulsifier and stabilizer agent. In addition, it is also used in the paper and cosmetic industries. In the present study, we performed a molecular characterization based on chemical composition and physicochemical properties to understand the properties behind TG applications. TG was extracted and purified from Tara seeds distributed in different ecoregions of Bolivia. The monosaccharide composition analysis was determined by high-performance anion-exchange chromatography/pulsed amperometric detection (HPAEC-PAD). At the same time, their molecular characteristics, such as molar mass, root-mean-square radius, hydrodynamic radius, conformation, and densities, were studied by asymmetrical flow field-flow fractionation coupled to multi-angle light scattering refractive index (AF4-MALS-dRI), also the specific refractive index increment (dn/dc) was determined for the first time using AF4 for TG. The results revealed that the gum samples are galactomannans composed of mannose (Man) and galactose (Gal) in a ratio of 3.37 (Man/Gal), with an average molar mass range from 2.460 × 10^7^ to 3.699 × 10^7^ Da, distributed in a single population. The root-mean-square radius range from 260.4 to 281.6 nm, and dn/dc is 0.1454. The Kratky plots based on 14 scattering angles indicated that the conformation of all samples corresponds to random coil monodisperse, while their gyration radius/hydrodynamic radius ratio (ρ) is high. All these results suggest that the chains have a low branched density, consistent with the Gal/Man composition. To the best of our knowledge, we report for the first time an integrated physicochemical study of TG relevant to developing emulsifier and stabilizer formulations.

## Introduction

Gums are colloquially called macromolecules with colloidal properties, including complex polysaccharides. These natural gum polysaccharides (GPs) are derived from various sources, such as plant seed endosperm (Tara gum and Guar gum), plant exudates (Tragacanth), shrubs or trees (Gum Arabic, Garaya gum, Cashew gum), algae extracts (agar), bacteria (xanthan gum), animal sources (chitin), and others^1^. These compounds are widely used as agents of innovation in the food and pharmaceutical industries because of their viscosity-enhancing, gelling, emulsifying, coating, thickening, and stabilizing properties^2^.

TG is composed mainly of galactomannans, which can form aqueous solutions (hydrocolloids) with high viscosity without forming gel^3^. TG is a healthy alternative for low-fat and low-calorie food products, and its demand has intensified globally, mainly because it is utilized as a thickening agent and stabilizer in food products. TG properties are comparable to that of carob beans and Guar gum^4^. The principal uses of galactomannans are in dairy products (in thickening desserts, and particularly in sorbets, ice creams, and low-energy fat substitutes), fruit-based water gels, powdered products (desserts and hot milk puddings), bakery goods (icings and cake mixes), coffee whiteners, baby milk formulations, seasonings, sauces and soups, tinned meats, and frozen and cured meat foods^5^. Indeed, regulatory authorities recognize TG as safe for consumption when used within the recommended limits. The U.S. Food and Drug Administration (FDA) consider TG a Generally Regarded as Safe (GRAS) ingredient, and the European Union approved it for use in the European Union as a food additive (E417).

Despite numerous studies investigating its composition and rheological properties, characterization at the molecular level of TG remains relatively limited. However, advanced analytical techniques such as High-Performance Anion Exchange Chromatography/Pulsed Amperometric Detection (HPAEC-PAD) and Asymmetric Flow Field Flow Fractionation (AF4) have paved the way for significant advances. AF4 has mild separation techniques and a broad working range (from 10^3^ KDa to 10^9^ Da molecular weight); it is primarily being used to measure particle size, polydispersity, and physical stability of various systems, such as bio-macromolecules and nanoparticles. In comparison with size-exclusion chromatography (packed column), AF4 (open channel) allows separation while preserving labile structures^6^. Combining these state-of-the-art methods offers the means to determine the monosaccharide composition and various physicochemical attributes of water-dispersed macromolecules. Such attributes include molecular weights, conformations, and other relevant factors crucial to effectively using TG as emulsifier and stabilizer.

In brief, AF4 is a comprehensive physical technique specially designed to separate macromolecules. Like chromatography, it is an elution technique, but in this case, the analyte retention occurs by applying fields that can carry the solute to regions of lower flux and there is no interaction with the separation membrane. The applied field gives more precise control than the two-phase distribution used for the same purpose in chromatography^7^. The separation occurs in a channel consisting of two long narrow blocks bolted with a spacer in between. The flow within this thin channel is laminar, with a steep parabolic flow profile driving particle separations. At the bottom is a semi-permeable membrane supported by a frit. The membrane is permeable to the solvent but not the analyte; this essential function is ensured by selecting the appropriate membrane pore size, expressed as a molecular weight cutoff (MWCO) ranging from 1 to 100 kDa^8^.

The molecular weight and specific refractive index increment are parameters of interest for characterizing the size and conformation of macromolecules, free or aggregates, in solution or suspension. The average molecular weight (Mw) takes into account the molecular weight of a chain in determining contributions to the molecular weight average^9^. While the dn/dc value describes the change in the refractive index of a polymer solution concerning the concentration of the solute, determining the dn/dc is essential for the absolute characterization of the molar mass by AF4^10^. dn/dc is defined as the slope of the dependence of the refractive index of polymer solution on its concentration^11^.

Although the molecular weight of TG was previously determined by different techniques such as gel permeation chromatography^12,13^, AF4 separates multimeric polysaccharide chains and aggregates based on their diffusion coefficient and it’s a physical separation technique which means that there is no interaction at any point, comparing AF4 with size exclusion chromatography in AF4 there is no stationary phase which reduces the potential loss of analytes from adsorption or shear-induced degradation^14^. This allows the determination of a broad distribution of molecular weights.

The samples used in the present study are from trees that grow in very particular environmental conditions, such as high altitude above sea level and dry soils compared to other production zones. These conditions showed to influence the composition of the pods in terms of total tannins, which is the key factor for the global market that requires a total tannin content of at least 48%, and the samples showed higher values (70–85%)^15^, for that reason it is also important to characterize the molecular properties of TG from the same region. In the present study, we investigate the monosaccharide composition by HPAEC-PAD, the specific refractive index dn/dc, the molecular weight, root-mean-square radius (r_rms_), hydrodynamic radius (r_h_), and conformation using Kratky plots and the determination of the dn/dc value of a Bolivian TG using the AF4 technique.

## Materials and methods

### Reagents

Bovine serum albumin (BSA), Sodium Chloride (NaCl) Potassium Hydroxide (KOH), Sodium Hydroxide (NaOH) gum Arabic, Sodium Nitrate (NaNO_3_), Sulfuric acid (H_2_SO_4_), standards of galactose, glucose and mannose were purchased from Sigma-Aldrich (St. Louis, MO, USA). Pure water was obtained from a Milli-Q system (Milli-pore Corp., Billerica, Massachusetts, USA).

### Sample collection

Tara pod samples were kindly supplied by an NGO named “Société de Coopération pour le Développement International” (SOCODEVI), in July 2019. The samples were collected from three different locations in the valley of Chuquisaca, Bolivia: Padilla 19°18′ S 64°18′ W (sample 1), Redención 64°8′52.17′′ W (sample 2), and Tomina 64°27′44″ W (sample 3), where the main production of Tara takes place.

### Gum extraction

TG was extracted from the endosperm of Tara seeds using a thermal–mechanical method^16^. The seeds were subjected to a thermal shock, heating at 170 °C for 5 min and cooling down to room temperature (20 °C). Then the seeds were grounded for a few seconds obtaining the seed shell, the endosperm, and the germ. The endosperm was separated mechanically and then suspended in hot water (70 °C). The suspension was filtered while hot. Then, the gum was precipitated by adding pure ethanol. The gum-obtained as a white solid- was dried at room temperature overnight.

### Total protein content

Protein content was determined by the Flash Dynamic Combustion method (modified Dumas method). This procedure consists of a complete sample combustion within a high-temperature reactor, followed by accurately and precisely determining the elemental gases produced. The process is rapid, around 6 min for a sample characterization. Total protein content was measured using FlashEA 1112 NC Analyzers from Thermo Fisher Scientific USA. The sample, 24.3 mg of TG, was weighed in a tin capsule and introduced into the combustion reactor via the autosampler, ensuring its complete combustion.

### Monosaccharides composition

Monosaccharide composition analysis was performed as described in previous works with slight modifications^17,18^. First, 19.7 mg of TG were subjected to acid hydrolysis with 300 µL of 72% H_2_SO_4_ and heating at 30 °C for 1 h with stirring. Afterwards, the pre-hydrolyzed acid solution was diluted to 4% H_2_SO_4_ by adding ultra-pure water (Milli Q) and heated at 100 °C for 1 h with stirring (Seaman Hydrolysis). Then the solution was cooled down in ice, and the pH was adjusted to 6 by adding 0.2 N NaOH. Finally, the solution was diluted 100 times with Milli Q water and filtrated through 0.2 μm membrane filters for further analysis. HPAEC-PAD (Sunnyvale, CA, USA) analysis was performed using a PA-20 column with 0.75 mM NaOH mobile phase at 0.5 mL/min flow for 30 min. Standards of galactose, glucose and mannose were prepared in Milli Q water in concentrations ranging from 0.5 to 20 μg/mL.

### Fourier transform infrared analysis

Fourier transform Infrared Spectrometry analysis was performed by placing a small portion of TG (without any previous treatment) in the diamond window of a Thermo Scientific Nicolet iS5 (USA) spectrometer. The data was acquired in the spectral region 700–4000/cm with a 4/cm resolution applying 16 measured scans per sample and a data spacing of 0.964/cm. The used laser current was 3.76 mA at 30.1 °C.

### AF4 instrumentation

The AF4 flow control was regulated with an Eclipse Separation System AF4 (Wyatt Technology, Santa Barbara, CA, USA). Injection was handled with an autosampler and carrier flow was delivered with an isocratic pump (both from Agilent 1200 series Agilent Technologies, Waldbronn, Germany) equipped with a vacuum degasser. The system was coupled to Multi-Angle Light Scattering (MALS) (DAWN Heleos II, Wyatt Technology) and dRI (Optilab T-Rex, Wyatt Technology) detectors, both with an operational wavelength of 664 nm.

The AF4 separation was carried out on a 23 cm trapezoidal channel. A regenerated cellulose membrane was used for the accumulation wall, with a molecular weight cutoff of 10 kDa (Merck Millipore, Darmstadt, Germany). The nominal spacer thickness was 350 µm with a breadth of 2 mm at the outlet and 22 mm at the inlet.

From the 18 angles present in the MALS detector, only 14 scattering angles were used for the calculations: 20.7°, 29.6°, 37.5°, 44.8°, 53.1°, 61.1°, 70.1°, 80.1°, 90°, 99.9°, 109.9°, 120.1°, 130.4° and 140°.

#### AF4 method parameters

The mobile phase was an aqueous solution of 10 mM NaNO_3_, and solutions of 1 mg/mL TG were also prepared in 10 mM NaNO_3_.

The injection volume was 40 μL, leading to an injection mass of 40 µg. The injection flow was 0.2 mL/min for 1 min. Before elution, focus/relaxation was performed for 3 min with a flow of 1 mL/min. Elution started with a flow of 1 mL/min (Vc(o)), decreasing exponentially with a half-life time of 4 min (t_1/2_) (Eq. 1 below) for a total time of 40 min. After the elution, the channel was cleaned for five minutes without crossflow.

Exponential decay of the elution step is defined in Eq. (1) as it was used in gums by^19^.

1 $$Vc\left( t \right)~ = ~Vc\left( o \right)~e^{{\frac{{ln2}}{{t{\raise0.5ex\hbox{$\scriptstyle 1$} \kern-0.1em/\kern-0.15em \lower0.25ex\hbox{$\scriptstyle 2$}}}}~t}} ~$$

In Eq. (1) Vc(t) is the crossflow rate as function of time, t, after the elution mode starts.

#### AF4 data processing

The data was collected and analyzed with Astra software 6.1 (Wyatt Technology, Santa Barbara, CA, USA). MALS and dRI detectors were calibrated with BSA (bovine serum albumin) and NaCl, respectively, at room temperature. Both molecular weight and radius of gyration of TG were obtained from the combination of MALS and dRI detectors, applying the Berry model^20^ with a 2nd order fit with 14 scattering angles (from 20.7° to 140.0°), this model was chosen because it is useful when analyzing molecules with high r_rms_ (greater than 50 nm). The dn/dc experimental value used was 0.1454 ml/mg (section “Specific refractive index increment (dn/dc)”). The second virial coefficient was considered negligible.

#### Determination of hydrodynamic radius

The hydrodynamic radius was calculated by using the FFFhydRad 2.1 MATLAB App^21^, applying the Stokes–Einstein equation:

2 $${\text{r}}_{{\text{h}}} = {\text{kT}}/{6}\pi \eta {\text{D}}$$

where D is the translational diffusion coefficient, ƞ the dynamic viscosity of the solvent, T is the absolute temperature, and k is the Boltzmann constant. For the calculations, the experimental channel thickness (w) was 286.4 µm, calculated using BSA with 6.6 nm of hydrodynamic diameter.

#### Determination of the specific diffractive index increment

TG’s dn/dc value was determined only using the detector (dRI). The samples were injected directly into the detector at five different concentrations (0.2, 0.4, 0.6, 0.8 and 1 mg/mL in 10 mM NaNO_3_). For the calculation, the differential refractive index results were plotted as a function of the concentration. Solutions at five different concentrations of NaCl and gum Arabic were used as controls to validate the method.

#### Conformation studies (Kratky plots)

The molecular conformation was estimated througth the Kratky plots^22–24^ from the angular variation of light scattering results. For this purpose, 12 MALS angles were taken into consideration, from 13.0° to 109.9° (corresponding to detectors 2–13). The data was collected from the MALS fractograms at the top of the peak for each sample (between 28 and 29 min of elution time).

The angular variation was calculated from the following equation:

3 $$\frac{{R_{\theta } }}{{K_{c} }} = MP\left( u \right)$$

where *R*_*θ*_ is the Rayleigh ratio, *c* is the analyte concentration, *K* is an optical constant, *M* is the molar mass, and *P(u)* is the form factor, where *u* is the product of r_rms_ and the scattering vector *q*. This last one can be calculated from the following equation:

4 $$q = \left( {\frac{{4\pi n_{0} }}{{\lambda_{0} }}} \right)\sin \left( {\frac{\theta }{2}} \right)$$

where *λ*_*0*_ is the wavelength of the incident light (664 nm), *n*_*0*_ is the refractive index of the solvent, and θ is the angle variation between the scattered and the incident light.

The Kratky plots are obtained by plotting *P(u)·u*^*2*^ as a function of *u*. As a reference, different theoretical values were plotted for different conformations of polymer structures^23^.

#### Dynamic Light Scattering (DLS) analyses

Molecular weight (Mw) and hydrodynamic radius (r_h_) were also determined using Dynamic Light Scattering (DLS) to compare the results obtained by AF4-MALS-dIR and to assess the presence of aggregates. The experiments were conducted at 20 °C using a Zetasizer APS from Malvern Panalytical (Malvern Instruments, UK). The sample changer and measurement cell were temperature-controlled. Samples were analyzed in 9 replicates at a 90° scattering angle in 96-well plates, and the system was rinsed with 0.1 M NaOH and ultra-pure water (Milli-Q) between each measurement. The software version used was 7.02. The samples were dissolved in 10 mM NaNO3, the same as for AF4 and in water. In both cases, solutions ranging from 1 to 0.1 mg/mL were analyzed to assess any concentration-dependent changes.

### Statements for research involving plants

This study adheres to all institutional and national regulations of Bolivia, including the collection of materials. This work has received authorization and oversight from The Institute of Chemical Research (IIQ) at Universidad Mayor de San Andrés, a government-accredited scientific institution. This species, *Caesalpinia spinosa,* has a wide distribution and is not categorized by either IUCN or CITES regulations.

## Results

### Monosaccharides composition and protein content

Polysaccharide hydrolysis, followed by HPAEC-PAD analysis, revealed that TG is composed of mannose (Man) and galactose (Gal) in a Man/Gal ratio of 3.37, and glucose (Glc). No other monosaccharides were detected (Table 1). These results indicate that TG is in terms of monosaccharides composition mainly a galactomannan. Total protein content was determined by modified Dumas method giving 0.74% (w/w).

**Table 1:** Monosaccharide composition of TG in percentage determined by HPAEC-PAD.

Monosaccharide	%
Galactose	6.02
Glucose	4.03
Mannose	20.27

### Fourier Transform Infrared analysis

**Figure 1:**
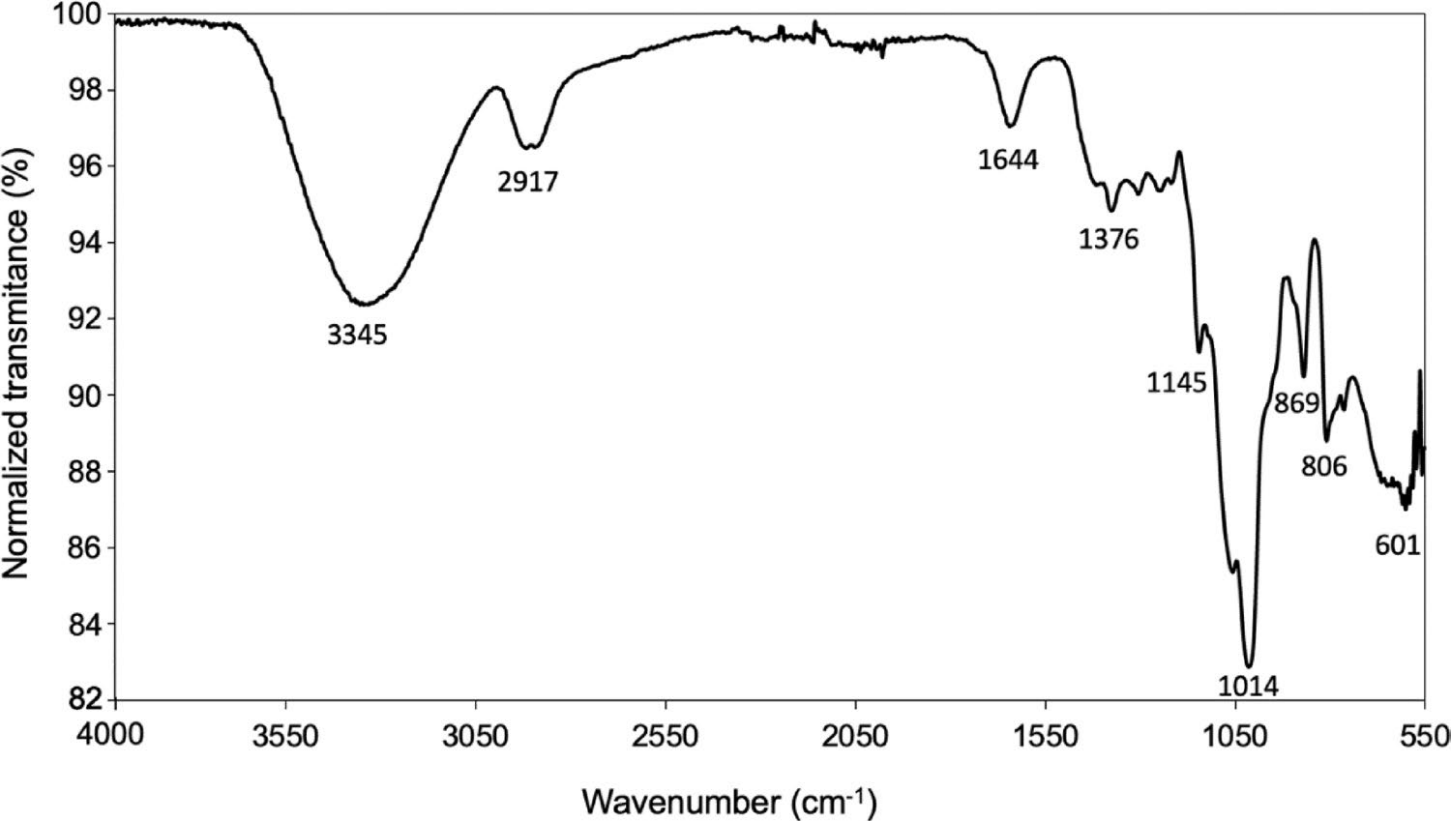
Fourier transform infrared spectra of TG.

The Fourier Transform Infrared analysis, depicted in Fig. 1, indicates a polysaccharide. The peak at 3345.76/cm corresponds to hydroxyl (OH) group stretching. At 2917.19/cm, the asymmetric extension of the carbon-hydrogen (C–H) bonds is observed, while the peak at 1376.3/cm is likely associated with C–H bending vibrations. The 1145.21/cm and 1014.69/cm peaks are likely associated with stretching carbon–oxygen (C–O) bonds, further confirming the carbohydrate's structural nature as pyranose. The structural characteristics of galactomannans become apparent, with the peaks at 869.65/cm and 809.63/cm attributed to vibrations related to α-d-galactopyranose and β-d-manopyranose bonds, respectively.

### Specific refractive index increment (dn/dc)

The specific refractive index increment enabled the comprehensive determination of the molecular characteristics of TG. The method was validated by determining the dn/dc for reference substances, NaCl and gum Arabic, for which the values are known. Thus, the results showed the same values previously reported for the reference substances, demonstrating the method’s reproducibility and, consequently, validating the dn/dc value obtained for TG (Table 2). It’s important to note that there is no previously reported reference value for TG, making this determination a novel contribution.

**Table 2:** Specific refractive index (dn/dc) determined at a wavelength of 658 nm, values expressed ± standard deviation.

Molecule	Experimental dn/dc	Reference dn/dc values
NaCl	0.1746 ± 0.0012	0.174^25^
Gum Arabic	0.1406 ± 0.0007	0.141^26^
Tara gum	0.1454 ± 0.0014	Present work

### Determination of the molar mass and size of Tara gum

**Figure 2:**
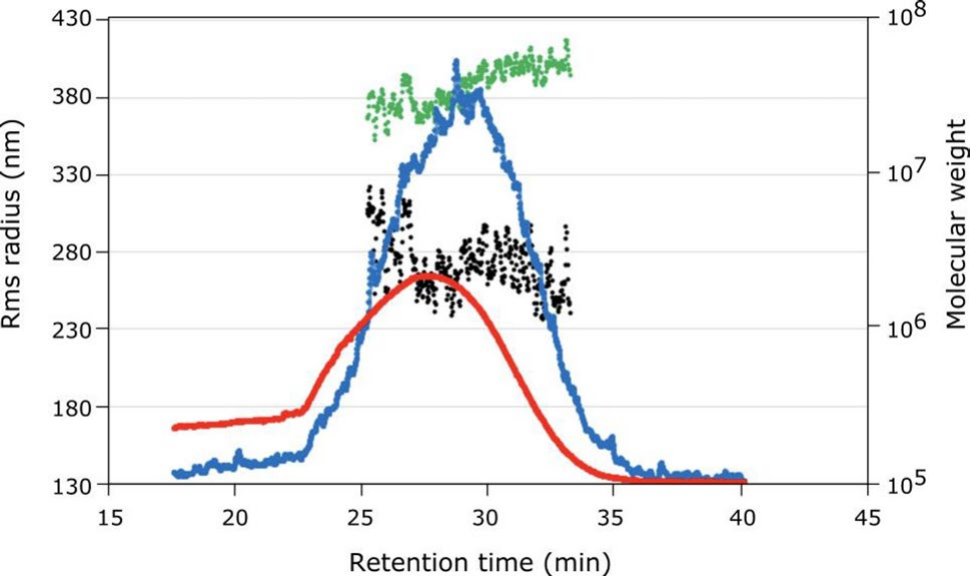
AF4 MALS-DRI fractograms of TG. MALS signal at 90° scattering angle (blue trace) and DRI signal (red trace). Molar mass (black trace) and the rms radius (green trace) vs. retention time of the sample (min).

The AF4 technique was employed to determine galactomannan’s molecular weight and size distribution. Figure 2 illustrates a typical AF4 MALS-DRI fractogram of TG galactomannan. The blue trace corresponds to the MALS signal, recorded at a scattering angle of 90°, while the red trace represents the DRI signal. The peak in the graph exhibits variations indicative of molar mass (as indicated by the black trace) and root-mean-square (r_rms_) radius (as indicated by the green trace).

For a comprehensive analysis, Table 3 provides data on the molecular weight (Mw), radius of gyration (r_rms_), and hydrodynamic radius (r_h_). These values were calculated using measurements from 14 scattering angles (MALS) ranging from 20.7° to 140°, collected at the peak elution time, as specified in Table 3.

**Table 3:** Molecular weight (Mw), radius of gyration (r_rms_), and hydrodynamic radius (r_h_) at the peak of the distribution for each sample, including standard deviation, along with the ratio (ρ = r_rms_/r_h_).

Sample	Time (min)	Mw (Da)	Average Mw (Da)	r_rms_ (nm)	Average r_rms_ (nm)	r_h_ (nm)	ρ = r_rms_/r_h_
1	28.878	(2.809 ± 0.140) × 10^7^	(2.694 ± 0.212) × 10^7^	253.2 ± 2.4	260.4 ± 4.2	43.804	5.780
2	28.744	(3.819 ± 0.509) × 10^7^	(3.699 ± 0.763) × 10^7^	281.7 ± 6.0	281.6 ± 8.7	44.809	6.287
3	28.794	(1.799 ± 0.252) × 10^7^	(2.460 ± 0.486) × 10^7^	231.1 ± 6.8	266.1 ± 8.2	45.052	5.129

Additionally, average molecular weight (Mw) and average radius of gyration (r_rms_) with standard deviation, obtained from measurements using 14 scattering angles (MALS).

The distribution of each sample, as depicted in Fig. 2, facilitated the calculation of each sample’s average molecular weight and radius of gyration. These results are presented in Table 3, showing that the average molecular weight (Mw) for the samples falls within a range of 2.460 × 10^7^ to 3.699 × 10^7^ Da, and the average radius of gyration (r_rms_) ranges from 260.4 to 281.6 nm. These values were derived from the AF4 MALS-DRI fractograms, which display the distribution of both Mw and radius.

Table 3 summarizes the data, including the time of elution, Mw, average Mw, r_rms_, average r_rms_, r_h_, and the ratio ρ = r_rms_/r_h_ for each of the three samples.

### Conformation studies of Tara’s galactomannan

The ratio r_rms_/r_h_ (ρ) serves as an indicator of polysaccharide conformation. While not providing precise conformational details, it offers an initial insight into the nature of the studied polysaccharide. Table 3 presents the calculation of this ratio (ρ) based on data obtained at the peak's apex.

**Figure 3:**
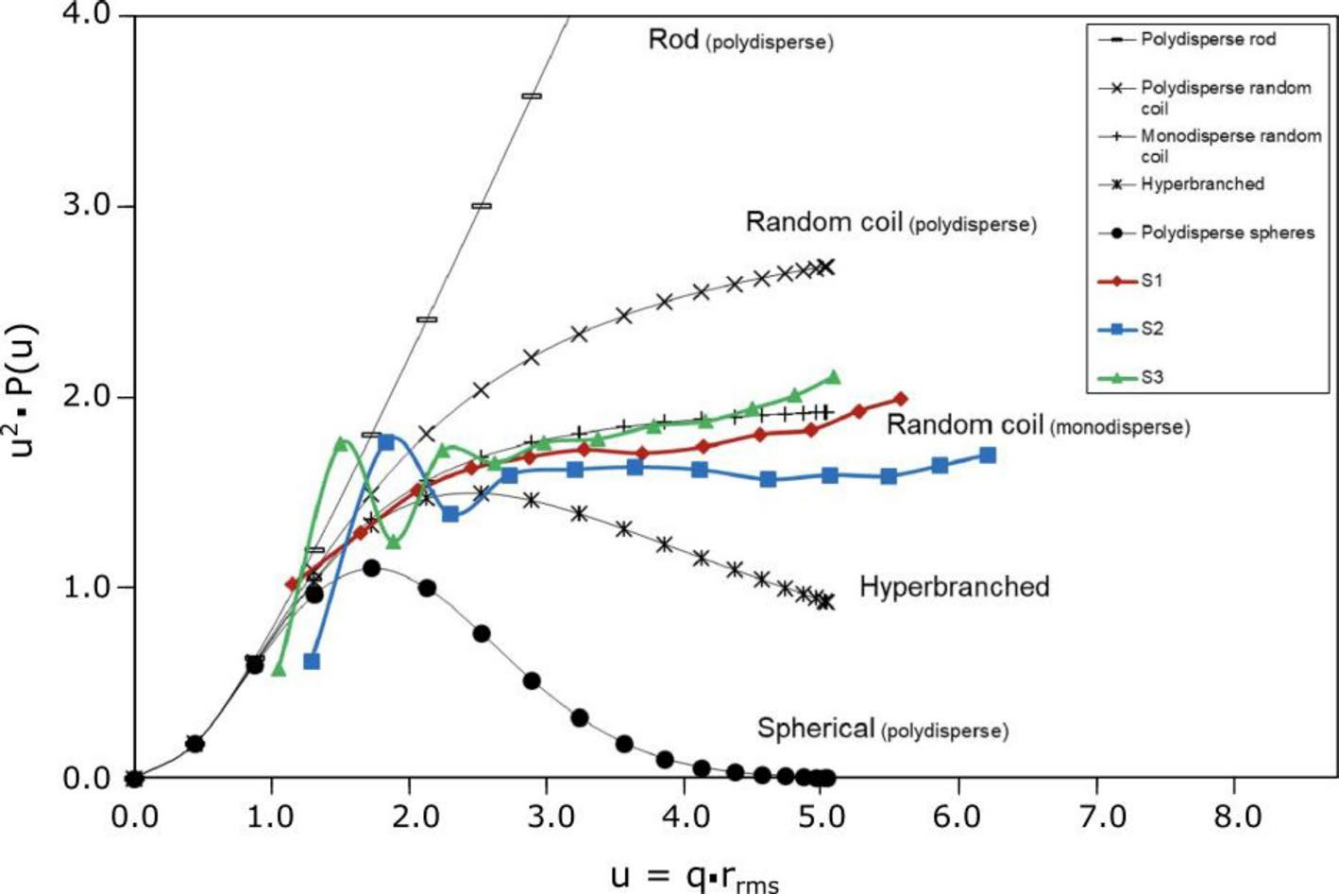
Kratky plots for 3 samples of TG obtained from MALS at 14 scattering angles.

Kratky plots were utilized for a more robust assessment of TG's conformation. Kratky plots enable a qualitative evaluation of unfolding and flexibility in samples, allowing for a more precise characterization of TG. Figure 3 shows the conformation of the three samples using these plots. Here, we observe a general trend toward monodisperse random coil conformation. Additionally, various polysaccharide conformations are provided as references within the same figure.

### Tara’s galactomannan DLS analysis

**Figure 4:**
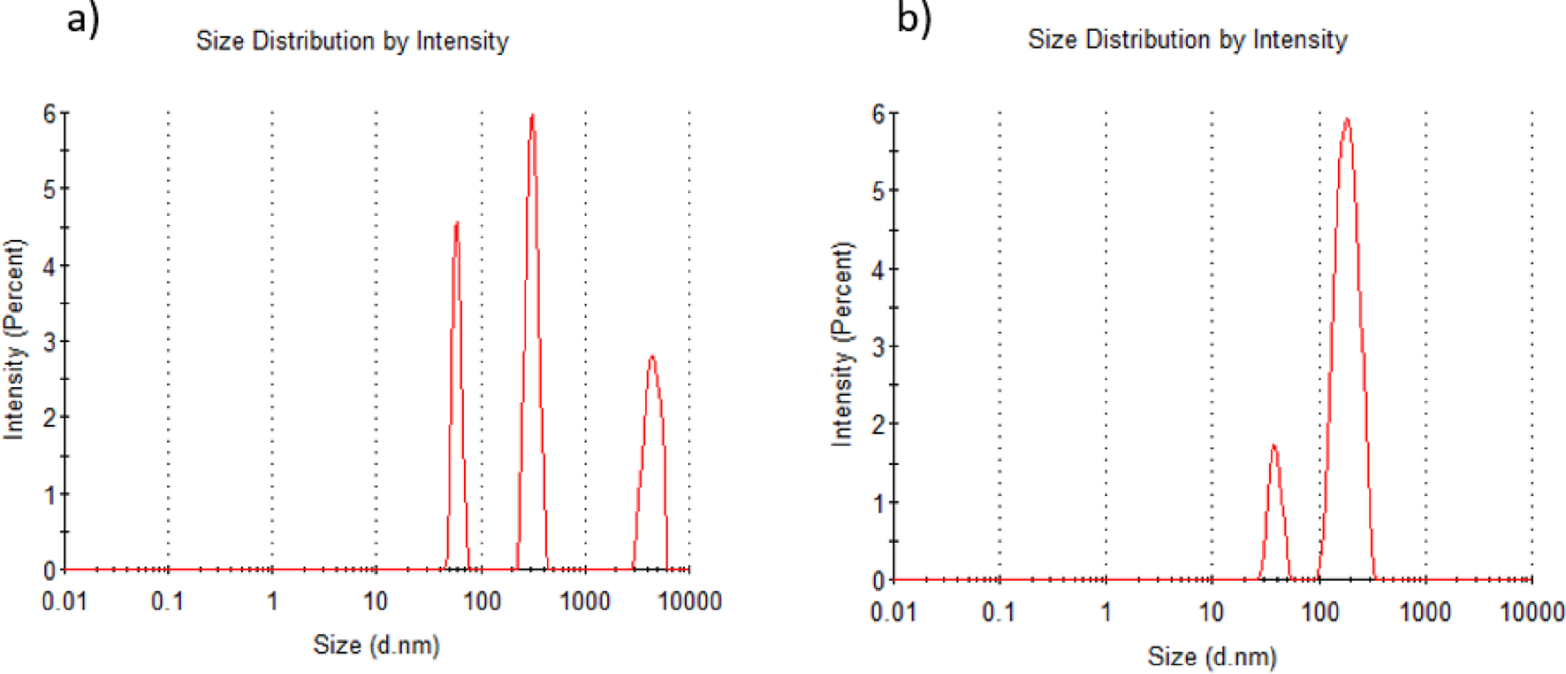
DLS size distribution by intensity of TG, (**a**) at 1 mg/mL concentration dissolved in NaNO_3_ and (**b**) at 1 mg/mL concentration dissolved on Milli Q water.

DLS analysis suggests that the samples have a significant proportion of aggregates. Figure 4 shows the size distribution by intensity of TG dissolved in NaNO_3_ (Fig. 4a) and in water (Fig. 4b), both solutions at a concentration of 1 mg/ml, which was the same concentration used in AF4. From the distribution, the Mw of the single structure (no aggregates) in NaNO3 solution is 9.14 × 10^3^ KDa but with an error of 12.36%, representing 87.4% of the mass distribution. At the same time, the Mw of the single structure in water is 3.44 × 10^3^ KDa with an error of 23.93%, representing 92% of the mass distribution.

## Discussion

TG is one of the three most important galactomannans in the food industry, and galactomannans from the same source can differ in terms of macromolecular characteristics between each other depending on their place of origin^27^. The analysis of Bolivian TG in the present study regarding monosaccharide composition confirms that the primary component of TG is galactomannan. The Man/Gal ratio for TG has been previously determined using various techniques such as NMR or FT-IR (Table 4). This study establishes the Man/Gal ratio as 3.37 through HPAEC-PAD, a powerful technique to separate and quantify monosaccharides. Notably, the ratios obtained using HPAEC are among the few that align closely with our findings. The sensitivity and enantiomer-separation capability of HPAEC enables a more precise determination of galactose and glucose than other techniques. The minor proportion of glucose (~ 4%) found in the TG could indicate the presence of glucans. The high Man/Gal ratio suggests that TG galactomannan can be classified as a thickening and gelling agent. Such galactomannans often exhibit limited solubility at lower temperatures and require heating for complete dissolution. The interaction between mannan chains enhances viscosity and may induce gel formation at high concentrations and lower temperatures^28^.

**Table 4:** Comparison of parameters determined in the present work with previous studies.

Parameter	Value	Method	References
Man/Gal ratio	3.37	HPAEC—PAD	Present work
	4.2	FT-IR and GC–MS	^40^
	2.9	HPAEC	^48^
	1.54–1.85	NMR	^29^
	3	HPAEC	^30^
Protein content	0.74%	Dumas	Present work
	< 3.5% (FAO/WHO)	–	^49^
	0.71%	NA2100 Nitrogen and Protein Analyzer	^30^
Specific refractive index (dn/dc)	0.1454	Refractive index detector from AF4 equipment	Present work
Molecular weight	(2.46–3.69) × 10^7^ Da	AF4 (average of distribution)	Present work
	9.14 × 10^3^ KDa	Dynamic Light Scattering (DLS)	Present work
	1519 × 10^3^ Da	Gel permeation	^50^
	2.18 × 10^6^ Da	Static Light Scattering(SLS)	^51^
	2.23 × 10^6^ Da	As viscosity average molecular weight	^30^
	1300–1500 KDa	Calculated from intrinsic viscosity	^29^
	3.7 × 10^6^ Da	Calculated from intrinsic viscosity	^52^
Radius of gyration	(231.1–281.7) nm	AF4 (average of distribution)	Present work
	(73–80) nm	Calculated from intrinsic viscosity	^29^
Hydrodynamic radius	(43.804–45.052) nm	AF4	Present work
	(77–85) nm	Calculated from hydrated volume	^29^
	63 nm	Calculated from intrinsic viscosity	^52^

The FT-IR analysis complements the chemical characterization of TG galactomannan and provides structural insights (Fig. 1). The overall spectra show typical peaks of polysaccharides, such as those corresponding to O–H stretching vibrations (hydroxyl groups) at 3345/cm and C–O–C stretching vibrations (glycosidic bonds) at 1014/cm. But it also has characteristic peaks of α-d-galactopyranose and β-d-manopyranose at 869.65/cm and 809.63/cm, respectively, well in line with the mannose and galactose composition determined by HPAEC-PAD. Similar spectra were reported previously^29^.

Little is known about the TG protein content. Only one study has reported 0.71% (w/w)^30^. We report here a similar value of 0.74% (w/w) (Table 4). According to the FAO and WHO, the protein should be lower than 3.5% (w/w).

The experimental specific refractive index increment (dn/dc), as explained in section “Determination of the specific diffractive index increment” is essential for the characterization of polymers since it is a value used in the molar mass determination. The dn/dc indicates the variation of the refractive index of a solution regarding the concentration of the solute. dn/dc obtained for TG in this work is 0.1454; it was shown that for polysaccharides in an aqueous buffer solution, the average dn/dc is 0.15^31^. It is also important to point out that this value for TG was not reported before.

The molecular weight for TG’s galactomannan showed a broad distribution, between 2.460 × 10^7^ and 3.699 × 10^7^ Da. AF4 technique offers several advantages over other separation methods, including label-free, gentle, rapid (< 1 h), highly reproducible, and efficient recovery of analytes. Most importantly, unlike other available techniques, AF4 can separate at high resolution (1 nm) and provide a large dynamic range of size-based separation^32^. AF4 can contribute to a better understanding of TG properties, enabling a direct determination of the properties. It is worth noting that most of these properties have also been determined previously, with the limitation of indirect techniques. One of the most used techniques to determine molecular characteristics is size exclusion chromatography (SEC), and it is also used for polysaccharides, however the range of molecular weight higher than 10^6^ Da of SEC is limited and the column absorption effect and shear effect of the stationary phase limit its application in polysaccharides, AF4 has gained a lot of interest lately for its benefits compared to other techniques^33^. In some cases, the molecular weight, radius of gyration (r_rms_) and hydrodynamic radius (r_h_) have been determined from intrinsic viscosity and hydrated volume (Table 4). Previous studies also suggested that the extraction time does not affect galactomannan's macromolecular parameters, but parameters such as yields and viscosity are affected by extraction time^27^.

Complementary to AF4, a DLS analysis of TG galactomannan was performed. DLS shown that a significant proportion of aggregates is formed both in water and NaNO_3_ water solution, when they are not subjected to any separation process before analysis, despite some authors reported that NaNO_3_ is used to avoid aggregates formation^34^. However, the galactomannan´s Mw in NaNO_3_ water solution was higher than in just water. Also, the Mw error deviations were lower in NaNO_3_ solution than in water. This could be interpreted as NaNO_3_ improving the identification of single structures because it was shown before that NaNO_3_ can reduce the degree of aggregation^35^, and also bigger structures are being considered (as we saw before in AF4). It was suggested before that separation processes can break down aggregates in galactomannans and they can be formed again during sample resting^27^. In DLS by its own there is no separation of the molecules, and the sensitivity of the molecular characteristics is around 20 times lower than MALS. It is also important to mention that DLS use a single fixed angle, while in MALS we used 14 different angles, leading to a more accurate measurement by AF4-MALS.

Hydrodynamic radius (r_h_) and molecular weight (M_w_) are not independent variables. When r_h_ increases, also M_w_ increases, but it depends on the shape of the polymer. In rigid rod structures, an increase in r_h_ is proportional to the M_w_ but up to the square root of M_w_ for those with a random coil^36^. In this case, TG samples have a random coil structure according to Kratky plots (Fig. 3). In polymers with the same r_h_ but different Mw, more solvents can enter the structure of those with lower Mw. For that reason, they will act as better thickening agents. More solvents can also enter when the chain is highly branched and lowly rigid^36^. Thus, linear polysaccharides have a higher viscosity than branched polysaccharides^37^. Extended molecular structures can entrain large amounts of water, increasing solution viscosity and, at higher concentrations, promoting depletion flocculation. In this way, extended-structure polysaccharides have a better capacity to stabilize emulsions than those with compact structures.

The ratio (ρ = r_rms_/r_h_) depends on the chain architecture. It was shown that the ratio ρ gives information about the conformation of the polysaccharide, in this case, if the value is higher than 2, the structure should be monodispersed^38^. All TG samples have high ρ values, higher than 2, suggesting that the conformation corresponds to monodisperse structures, which can also be confirmed in the Kratky plot (Fig. 2). It was also shown that higher values of ρ can be caused for a low branched density in the chain^39^, which suggests that TG has a monodisperse random coil structure with low branched density.

Ba et al.^40^ showed that the viscosity of TG solutions increases with an increase in their concentration, but both viscosity and elasticity are easily affected by the effect of shear stress. TG showed higher viscosity at a shear rate lower than 80 Hz. Viscosity is caused by the friction arising when molecules interact with each other. Perhaps the fraction with higher viscosity has more chain entanglement when the shear force is applied to the solution. For example, the conformational properties of gum Ghatti were analyzed, one of the fractions with a random coil conformation, less branched chain and lower molecular weight than the others, showed higher viscosity^38^.

Also, the solubility of a polymer in a given solvent can decrease as molecular weight increases, conformation can play an important role^12^. Some plant gums, including TG, are only partially soluble in water; it has been proved that water solubility of polysaccharides is enhanced by increasing the irregularities in their structure^41^. It was reported that a high molecular weight might be associated with superior emulsion stabilizing properties^42^, other good correlations between emulsion stability and average M_w_ of a well-known emulsifier (gum Arabic) were observed, first the 10% of the population of gum Arabic separated by gel permeation chromatography had the highest molecular weight, but it produced a more stable emulsion than the remaining 90% which had a lower molecular weight^43^, then another study^44^ reported that commercial gum Arabic weight-average Mw ≈ 6 × 10^5^ Da did not produce a stable emulsion at concentrations < 20%. But when the weight-average Mw was increased to a range of 1–2.5 × 10^6^ Da by a controlled maturation process, the emulsion stability was greatly increased under a similar condition, even at a 5% gum concentration.

In the food industry, TG is considered a good substitute of Guar gum and locust bean gum, mainly because its water solutions are neutral and highly viscous because they are soluble in cold water^45^, galactomannans have also been shown to enhance the water-holding capacity when added to food systems^46^. Some researchers also suggested that further studies are needed to understand aggregates' role in galactomannans' functional and physicochemical attributes, such as rheological behavior and solution characteristics^47^.

Several committees have already evaluated TG as a food additive based on its rheological properties and in vitro studies with animals due to its properties as a thickener, emulsifier, and stabilizer. Having the molecular characteristics determined for the first time with more accurate techniques, such as HPAEC-PAD and AF4, enables an understanding of these properties, and this work’s results can contribute to developing new formulations and products.

## Conclusions

A compressive characterization of TG samples from different regions of Bolivia was carried out using advanced instrumental techniques such as HPAEC-PAD and AF4. To our knowledge, the molecular weight distribution, specific refractive index, radius of gyration, hydrodynamic radius, and conformation of TG were determined for the first time using AF4 in this work. The radius and molecular weights differed for each sample, possibly due to their different geographical origins. Analysis of the Kratky plots showed the same conformation for random coil monodisperse polymer in all samples, while the gyration radius/hydrodynamic radius ratio and the Man/Gal ratio suggest that TG contains lowly branched polymers. All these molecular characteristics contribute to understanding TG properties as a thickener, emulsifier, and stabilizer and set the basis for developing new products.

## Data Availability

The datasets generated during and/or analyzed during the current study are available from the corresponding author on reasonable request.
